# The Diagnosis and Treatment of Diseases of the Rectum

**Published:** 1897-06

**Authors:** 


**The Diagnosis and Treatment of Diseases of the Rectum.** By William Allingham and Herbert W. Allingham. Sixth Edition. Pp. xvi., 485. London: Baillière, Tindall and Cox. 1896.

This work is well known, and is evidently useful to the profession. This edition maintains the reputation of its authors. The publishers' part has been well done; the printing of the exceedingly large type and the paper are both excellent, and the illustrations are sufficient. Messrs. Allingham aim at giving us a practical guide to the diseases of which they treat, and we think they have succeeded admirably. There is an excellent chapter on the methods of investigation and examination of rectal cases, and throughout the book the lines of treatment are described with a fulness and attention to detail that, without being dull, add very materially to its practical usefulness. Many valuable hints and suggestions will be found scattered through it, and there is no lack of formulæ and prescriptions for the benefit of the practitioner. The pathology of the various diseases is only described so far as it directly affects their treatment, and this gives a conciseness and unity to the whole that make it very easy reading. The account of the operative treatment of internal hemorrhoids suffers, we think, from overcrowding. Although the desire to do justice to all suggested methods of treatment is praiseworthy, yet we think the subject would have gained in clearness and practical value by a more personal and less encyclopædic method. The authors express themselves strongly in favour of the treatment by ligature; but while they evince a rather puzzling amount of tolerance for many other kinds of treatment, they are singularly cavalier in their summary rejection of that by the clamp and cautery, considering the high degree of favour it enjoys in many quarters. We confess ourselves greatly surprised at the grounds of their condemnation, and we think their experience of this method must have been singularly unfortunate. Whitehead's method is severely criticised and emphatically condemned.

A large amount of space is allotted to cancer and stricture of the rectum and their treatment, and on the whole the account is excellent. The advice given as to the choice between inguinal and lumbar colotomy seems to us illogical. The superiority of the left inguinal operation hardly needs pointing out and, the risk of infecting the peritoneum apart, it should be the method of election except when the sigmoid itself is affected in its upper or middle portions, or when the cancerous growth affects higher portions of the large bowel.

The subject of congenital malformation is ignored, although it would seem to hold a legitimate place in such a work. In spite, however, of the slight reservations we have made, we can cordially recommend the book to all who have to deal with rectal diseases.

